# Supplementary material for: Living with and managing type 1 diabetes in humanitarian settings: A qualitative synthesis of lived experience and stakeholder tacit knowledge
Source: PLOS Glob Public Health. 2024 Jun 21;4(6):e0003027. doi: 10.1371/journal.pgph.0003027 (PMC11192347; doi:10.1371/journal.pgph.0003027)
Supplement: S1 Text — (DOCX) [file pgph.0003027.s001.docx]

# **S1. Literature search strategy for Medline (OVID) database**

1. (("type 1" or "type one" or juvenile or sudden or brittle or childhood) adj4 diabet*).mp.

2. "T1D".mp.

3. Diabetes Mellitus, Type 1/

4. 1 or 2 or 3

5. ((conflict or fragile) adj4 (crisis or crises or setting*)).mp.

6. (humanitarian or war or wars or conflict affected or migrant* or refugee* or asylum seeker* or displaced person or displaced people or disaster victim* or natural disaster* or flood* or earthquake* or hurricane* or "post conflict" or armed conflict*).mp.

7. exp armed conflicts/ or warfare/ or war exposure/

8. disaster victims/ or exp "emigrants and immigrants"/ or refugees/

9. 5 or 6 or 7 or 8

10. 4 and
