# Supplementary material for: Living with and managing type 1 diabetes in humanitarian settings: A qualitative synthesis of lived experience and stakeholder tacit knowledge
Source: PLOS Glob Public Health. 2024 Jun 21;4(6):e0003027. doi: 10.1371/journal.pgph.0003027 (PMC11192347; doi:10.1371/journal.pgph.0003027)

# **S2 Images of manual analysis process- establishing codes, sub-themes, and themes**

Fig 1: Braun and Clarke’s 6-step RTA process: Step (1) Becoming familiar with data and (2) Generation of initial codes (green).


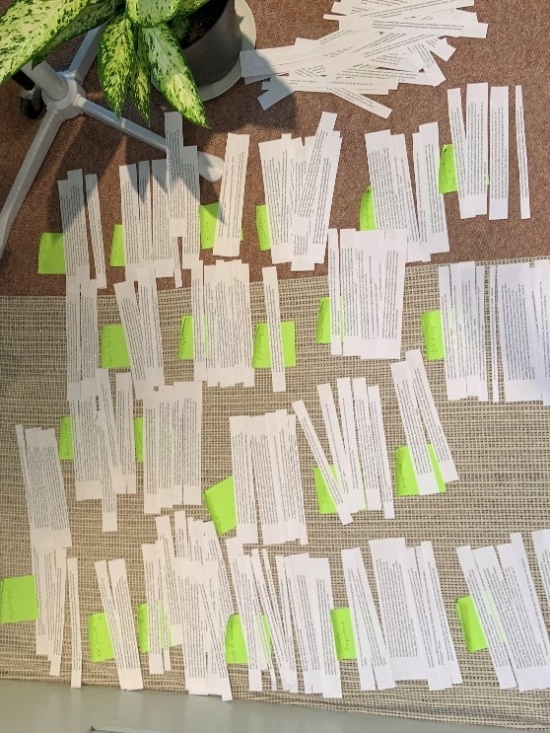


Fig 2: Braun and Clarke’s 6-step RTA process: Step (3) Initial construction of themes (pink).


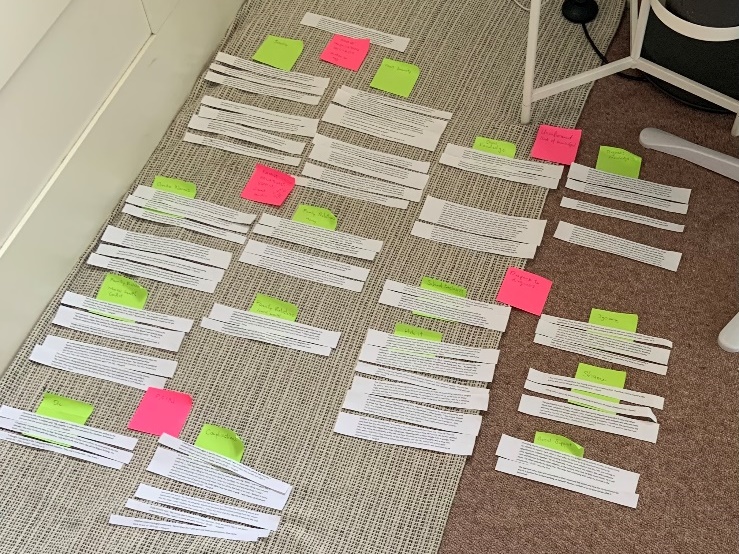


Fig 3: Braun and Clarke’s 6-step RTA process: Step (4) Revision of themes and (5) Updated theme names and definitions.


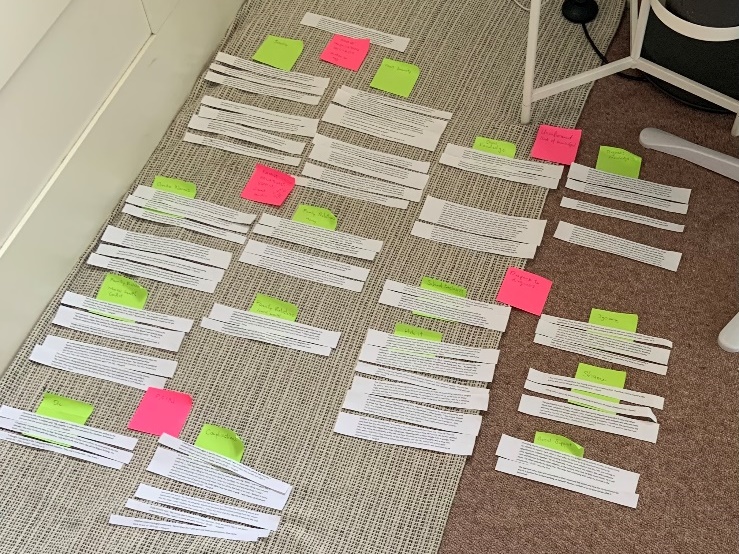

Supplement: S2 Text — (DOCX) [file pgph.0003027.s002.docx]
