# Supplementary material for: Living with and managing type 1 diabetes in humanitarian settings: A qualitative synthesis of lived experience and stakeholder tacit knowledge
Source: PLOS Glob Public Health. 2024 Jun 21;4(6):e0003027. doi: 10.1371/journal.pgph.0003027 (PMC11192347; doi:10.1371/journal.pgph.0003027)
Supplement: Attachment — Submitted filename: April 14_PLOSGlobaLHealth_SH.docx [file pgph.0003027.s004.docx]

April 14, 2024

Dear Editors,

Thank you for the opportunity to review the article “Living with and managing type 1 diabetes in humanitarian settings: a qualitative synthesis of lived experience and stakeholder tacit knowledge.” This is very well written article that uses qualitative synthesis methods to provide a review of the challenges faced by persons with type 1 diabetes in humanitarian settings. While I have minor suggestions for considerations, I believe this work provides an important contribution to the literature highlighting the special considerations that are needed for persons living with type 1 diabetes. The most important point to consider is adding information on how the results of this synthesis compare to what is known about the management of type 2 diabetes in humanitarian settings. A strong case is made as to why it is important to consider type 1 diabetes separately but does not close the loop by describing how the findings differ from what is known for type 2 diabetes in humanitarian settings. Highlighting the differences between type 1 and type 2 diabetes in this context will emphasize the importance of this work.

**Abstract:**

The last sentence should be adjusted to better reflect the findings of the article. The terms “structural and international levels” are vague and difficult to understand and relate to what is presented in the preceding abstract paragraph.

**Introduction:**

Page 3, paragraph 2, last sentence. “Forgone daily wage associated with…” is not clear and authors should clarify what they are trying to get at – the need to seek care means they lose a day of work?

Page 3, Paragraph 3, first sentence. “Barriers and challenges” is redundant.

Page 4, Paragraph 1. I would be careful to over-generalize. There are persons with type 2 diabetes who without insulin for several days will similarly become extremely sick and become at risk for hyerosmolar coma.

Page 5 paragraph 1, first sentence. The word “manifestation” is not correct as it implies incidence. Whereas the following sentence discusses diabetes control (with level of hemoglobin A1c).

Page 6 paragraph 2. First sentence. Missing word “people” before living.

**Methods and Results** are well written, and I especially liked Figure 2 as a depiction of themes, subthemes and illustrative quotes.

**Discussion**

Also well written with clear delineation of recommendations. As noted previously my one strong recommendation would be to specifically identify how this differs from review of challenges and recommendations for type 2 diabetes.
