# Supplementary material for: Living with and managing type 1 diabetes in humanitarian settings: A qualitative synthesis of lived experience and stakeholder tacit knowledge
Source: PLOS Glob Public Health. 2024 Jun 21;4(6):e0003027. doi: 10.1371/journal.pgph.0003027 (PMC11192347; doi:10.1371/journal.pgph.0003027)
Supplement: Attachment — Submitted filename: Point by Point Response.docx [file pgph.0003027.s005.docx]

**Point by Point Response**

**Editor**
The manuscript has been evaluated by two reviewers, and their comments are available below. Although both reviewers are positive about your manuscript, they have several requests for modifications. Please see the comments below and in the attached documents. Could you please carefully revise the manuscript to address all comments raised?

Reviewer 1 notes that the discussion section consists largely of recommendations and suggests that the section could "either be left as it is, or it could be split into a discussion section (reflecting on the implications of the findings for practice), and a recommendations section (framed around the three recommendations that are presented)." Like reviewer 1, I do not have a strong opinion, and so leave the suggestion to the authors for consideration.

**Response and action:** Thank you for your prompt and constructive input on this submitted manuscript. We are happy to change the section header from discussion to recommendations as suggested.

**Reviewer 1**

This is an interesting and relevant manuscript; the topic is of increasing importance in humanitarian healthcare, especially since recent emergencies (Ukraine, Gaza) have highlighted the challenges associated with Insulin provision during the first 30 days of the emergency response. Bringing together perspectives of people with lived experience enriches the policy discussions in the build up to the UN high level meeting on NCDs in 2025. The manuscript is well written, the analytic frame is justfied, and the final categorisation of themes makes sense. The findings are presented clearly and I believe will resonate well with other practitioners working in this field.

I have made a number of minor comments in the manuscript itself. My only substantial comment is about the discussion. The discussion as presented is more accurately described as a set of policy recommendations, supported by the findings of the study and by a broader literature on this subject. It reads well, and personally speaking I find it a helpful and powerful way to conclude the manuscript. But from a teminological point of view, it would be more accurately referred to as 'Recommendations' rather than 'Discussion'. In the end, it could either be left as it is, or it could be split into a discussion section (reflecting on the implications of the findings for practice), and a recommendations section (framed around the three recommendations that are presented). I do not have a strong opinion on this, I would suggest this is an editorial decision.

**Response and action:** Thank you for your generous inputs and suggestions. We have marked your inputs in the tracked-changed manuscript and addressed these mainly language edits and clarifications directly. We have also taken on the suggestion of using the term recommendations to replace discussions as a section header.

**Reviewer 2**

Thank you for the opportunity to review the article “Living with and managing type 1 diabetes in humanitarian settings: a qualitative synthesis of lived experience and stakeholder tacit knowledge.” This is very well written article that uses qualitative synthesis methods to provide a review of the challenges faced by persons with type 1 diabetes in humanitarian settings. While I have minor suggestions for considerations, I believe this work provides an important contribution to the literature highlighting the special considerations that are needed for persons living with type 1 diabetes. The most important point to consider is adding information on how the results of this synthesis compare to what is known about the management of type 2 diabetes in humanitarian settings. A strong case is made as to why it is important to consider type 1 diabetes separately but does not close the loop by describing how the findings differ from what is known for type 2 diabetes in humanitarian settings. Highlighting the differences between type 1 and type 2 diabetes in this context will emphasize the importance of this work.

**Response and action:** We have indicated the how the differences between T1D and T2S can be accounted for in programs- specifically the need to program for younger populations and their families, creating awareness in communities and capacity within health programs and facilities to detect and manage T1D.

Abstract:
The last sentence should be adjusted to better reflect the findings of the article. The terms “structural and international levels” are vague and difficult to understand and relate to what is presented in the preceding abstract paragraph.

**Response and action:** This has been edited to read: *Targeted family and community-based solutions are urgently required, alongside systemic reforms and international collaboration to enable better T1D coping and management in humanitarian settings.*

Introduction:
Page 3, paragraph 2, last sentence. “Forgone daily wage associated with…” is not clear and authors should clarify what they are trying to get at – the need to seek care means they lose a day of work?

**Response and action:** This point has been expanded to clarify the message- that especially in the context of daily wage employment, health-seeking can mean foregoing the wages earned for the day.

Page 3, Paragraph 3, first sentence. “Barriers and challenges” is redundant.

**Response and action:** This is edited.

Page 4, Paragraph 1. I would be careful to over-generalize. There are persons with type 2 diabetes who without insulin for several days will similarly become extremely sick and become at risk for hyerosmolar coma.

**Response and action:** We absolutely agree, and have clarified to suggest that milder forms can be managed with oral medications and may not need insulin, but the same cannot be said about T1D.

Page 5 paragraph 1, first sentence. The word “manifestation” is not correct as it implies incidence. Whereas the following sentence discusses diabetes control (with level of hemoglobin A1c).

**Response and action:** This has been edited.

Page 6 paragraph 2. First sentence. Missing word “people” before living.

**Response and action:** This has been edited.

Methods and Results are well written, and I especially liked Figure 2 as a depiction of themes, subthemes and illustrative quotes.

Discussion
Also well written with clear delineation of recommendations. As noted previously my one strong recommendation would be to specifically identify how this differs from review of challenges and recommendations for type 2 diabetes.

**Response and action:** This is an important observation, and we have ‘closed the loop’ in the recommendations and conclusion sections by highlighting how the nuanced differences for T1D can be managed, but also how there are indeed broad similarities in the overarching actions required- such as sustainable financing for NCDs.
